# Effects of Long-Term Multimodal Psychosocial Treatment on Antipsychotic-Induced Metabolic Changes in Patients With First Episode Psychosis

**DOI:** 10.3389/fpsyt.2018.00488

**Published:** 2018-10-16

**Authors:** Martina Rojnic Kuzman, Dina Bosnjak Kuharic, Ivana Kekin, Porin Makaric, Zoran Madzarac, Ana Koricancic Makar, Suzan Kudlek Mikulic, Zarko Bajic, Petra Bistrovic, Dora Bonacin, Zeljka Vogrinc

**Affiliations:** ^1^Department of Psychiatry, University Hospital Centre Zagreb, Zagreb, Croatia; ^2^Zagreb School of Medicine, University of Zagreb, Zagreb, Croatia; ^3^Psychiatric Hospital Vrapce, Zagreb, Croatia; ^4^Biometrika Healthcare Research, Zagreb, Croatia; ^5^Department of Laboratory Diagnostics, University Hospital Centre Zagreb, Zagreb, Croatia

**Keywords:** antipsychotics, schizophrenia, first episode psychosis, metabolic abnormalities, weight gain, psychosocial intervention, day hospital

## Abstract

**Background:** Antipsychotic-induced weight gain and metabolic abnormalities are one of the major challenges in the treatment of psychosis, contributing to the morbidity, mortality and treatment non-adherence. Different approaches were used to counteract these side effects but showed only limited or short-term effects. This study aims to analyse the effects of a long-term multimodal treatment program for first episode psychosis on antipsychotic-induced metabolic changes.

**Methods:** We enrolled 71 patients with first episode psychosis treated at the Zagreb University Hospital Centre from 2016 until 2018. Participants were assigned to one of the two groups: day hospital program vs. treatment as usual (TAU). Outcomes were: body weight, blood glucose, lipids and cholesterol, psychopathology and global level of functioning during the 18-months follow-up.

**Results:** Although the TAU group gained more weight and had higher increase of blood glucose, while the day hospital group had a higher increase in total cholesterol at 18th month follow-up, after the adjustment for age, gender and baseline measures, the type of treatment was not significantly associated with any of the primary outcome measures. Patients' psychopathology measures significantly decreased and their functional level significantly increased at month 18th in both groups.

**Conclusion:** While both types of treatment were effective in reducing psychopathology and restoring the patients' level of functioning, both were relatively ineffective in counteracting antipsychotic-induced metabolic abnormalities and antipsychotic-induced weight gain.

## Introduction

The second generation antipsychotics (SGA) are considered as the first line treatment for schizophrenia spectrum disorders. Unfortunately, most SGA are associated with a significant weight gain causing subsequent development of metabolic syndrome and increasing the risk of cardiovascular morbidity and mortality (1). In addition, significant weight gain may cause non-adherence to medication (2) and subsequently lead to relapses (3). Risk factors for antipsychotic-induced weight gain and metabolic abnormalities include polypharmacy (4), olanzapine or clozapine monotherapy (5), female gender, lower initial body mass index (6), positive treatment response (7) and family history of diabetes (8). The highest increase of weight gain is seen particularly during the first few months of treatment (9). Different strategies were used to counteract these side effects, such as changes of medication regimes (e.g., switch to antipsychotics with a lower propensity to cause weight gain, addition of drugs that may suppress appetite) (10, 11), psychoeducation (12) and exercise (13). However, in the clinical practice,antipsychotic-induced metabolic disturbances still remain one of the most important obstacles for effective treatment. During the last 20 years, multimodal programs for treatment of patients with first episode psychosis have been developed worldwide (14). These services usually incorporate a set of psychopharmacological and psychosocial approaches following individualized treatment plans. Patients with the first episode psychosis treated in our hospital are offered this kind of treatment through the day hospital setting. As this multimodalprogramme incorporatesstructured psychoeducation on healthy lifestyle and side effects of medication and unstructured exercise, we hypothesized that it may have advantage over treatment as usual (TAU) in counteracting antipsychotic-induced metabolic changes. Thus, we aimed to analyze the effects of the multimodal treatment of first episode psychosis on the antipsychotic-induced metabolic changes over an 18-months period. In addition, we explored the relationship of patients' psychopathology and functioning and the type of treatment.

## Methods

### Participants and protocol

This study was nested within the prospective cohort study: “Biomarkers in schizophrenia-integration of complementary methods in longitudinal follow up of first episode psychosis patients.” The sample consisted of patients admitted to Zagreb University Hospital Centre (ZUHC), between 2016 and January 2018. Adult patients with the first psychotic episode pertaining to the schizophrenia spectrum diagnosed according to the research criteria of the International Classification of Disorders (ICD-10) (15) by the consensus of two experienced psychiatrists, were recruited to participate in the study. Exclusion criteria included the presence of somatic disorders/status, and use of medication and other substances with possible effects on metabolic features (diabetes, hypercholesterolemia, pregnancy or lactation, antidiabetic drugs, hypolipemics), and psychotic disorders due to organic causes. All patients treated at the ZUHC for first acute episode psychosis during the study period meeting those criteria were invited to participate in the study. After the patients reached the subacute phase of psychosis (usually in the first few weeks of antipsychotic treatment), they chose one of the two treatment groups according to their preference: day hospital treatment (which included a combination of pharmacological approach and psychosocial approaches (group psychotherapy, social skills training, metacognitive training, creative workshops, family therapy, and psychoeducation including topics on healthy lifestyle, antipsychotic-induced metabolic changes, diet encouragement and unstructured exercise in a closed group, three times a week, but decreasing in the number of weekly sessions over 12 months) or TAU (short outpatients visits usually once a month). The patients were followed-up for 18 months and assessed at baseline and month 18th for sociodemographic and clinical data, including data on lifestyle (smoking, drinking, drug use), metabolic parameters [blood glucose, total cholesterol, high density lipoprotein (HDL), low density lipoprotein (LDL), triglycerides], weight, psychopathology with Positive and Negative Syndrome Scale, PANSS (16), quality of life using the World Health Organization Quality of Life Assessment, WHOQOL-BREF (17) and level of functioning using the Global Assessment of Functioning, GAF (18). Primary outcome of the study were metabolic side- effects assessed at 18-month. Secondary outcome included patients' psychopathology assessed at 18-month.

The study protocol was approved by the Ethics Committee of the ZUHC. The study was executed in accordance with World Medical Association Declaration of Helsinki 2013.

### Statistical analysis

Changes from the baseline values to values at the last assessment were presented with absolute and relative changes and their 95% confidence intervals. Differences in primary and secondary outcome measures between the two groups were analyzed using multivariable analysis of covariance (MANCOVA), adjusted for age, sex and baseline measurements (metabolic and BMI, and psychopathology). As the assumptions for MANCOVA we tested linearity of correlations between dependent and independent variables and multivariate normality by inspection of scatter plots, non-existence of univariate outliers and homogeneity of regression slopes, and the equality of error variances by Leven's test. The statistical significance was set at *p* < 0.05. Statistical data analysis was performed using the R Core Team (21).

## Results

We recruited 71 patients in the study and treated with day hospital treatment (*n* = 21) or TAU control group (*n* = 50). Sociodemographic and clinical characteristics at baseline are given in Table 1. The reasons for not choosing daily hospital over TAU were as follows: significant travel distance from hospital (*N* = 25), being employed or regular student with obligations several days per week and thus unable to attend the sessions (*N* = 11), being older than the majority of group participants and do not felt as fitting the group (*N* = 5) and preferring the individual over group treatment (*N* = 10).

**Table 1:** Baseline participants' characteristics.

	**Day hospital (*****n*** = **21)**		**TAU (*****n*** = **50)**	
Male gender	12	(57.1)	33	(66)
Age (years), median (IQR)	24	(21–25)	25	(22–33)
Being single	18	(85.7)	38	(76)
Years of education, mean (SD)	14	(3.2)	14	(4.3)
Employed	3	(14.3)	11	(22)
Age of first contact with psychiatry, median (IQR)	23	(19–25)	25	(20–31)
Number of cigarettes/day, mean (SD)	10	(11)	8	(12)
Occasional drug use	11	(54.2)	21	(42)
Occasional alcohol use	1	(4.7)	4	(8)
**MAIN ANTIPSYCHOTIC**				
amisulpirid or aripiprazole	5	(23.8)	5	(10)
fluphenazine or haloperidol	2	(9.5)	8	(14)
clozapine, olanzapine, quetiapine	8	(38.1)	21	(42)
risperidone, paliperidone	6	(28.6)	16	(34)
More than one antipsychotic	10	(47.6)	21	(42)
Mood stabilizers	3	(14.3)	12	(24)
Anxiolytics/hypnotics	6	(28.6)	17	(34)
Anticholinergics	5	(23.8)	11	(22)
Antidepressants	3	(14.3)	4	(8)

Data are presented as n (%), unless stated otherwise.

*IQR, interquartile range; SD, standard deviation*.

During the study period, 12 patients from the TAU group (24%) were re-hospitalized due to relapses, all because of non-adherence to medication, and 3 patients from the day hospital group (14.2%). At the final assessment, patients in the day hospital group were treated with amisulpiride or aripiprazole (*n* = 8, 38.1%), clozapine/quetiapine /olanzapine (*n* = 5, 23.8%) or paliperidone/risperidone (*n* = 4, 19%), or stopped any antipsychotics (*n* = 4, 19%); a significant number of patients had at least one additional medication (another antipsychotic (*n* = 7, 33.3%), sedative/hypnotic (*n* = 1, 4.8%), mood stabilizer (*n* = 3, 14.3%), antidepressant (*n* = 6, 28.6%), 3 anticholinergic (*n* = 2, 9.5%)); patients in the TAU were treated with amisulpiride or aripiprazole (*n* = 15, 30%), haloperidol/fluphenazine (*n* = 3, 6%), clozapine/quetiapine/olanzapine (*n* = 13, 26%) or paliperidone/risperidone (*n* = 15, 30%), or stopped any antipsychotics (*n* = 4, 8%); a significant number of patients had at least one additional medication (another antipsychotic (*n* = 20, 40%), sedative/hypnotic (*n* = 6, 12%), mood stabilizer (*n* = 10, 20%), antidepressant (*n* = 9, 18%), 3 anticholinergic (*n* = 7, 14%)).

### Metabolic outcomes over the longitudinal follow-up

Changes of the metabolic features and BMI, as well as changes in symptoms over the 18-months longitudinal follow-up are given in Table 2. The associations of type of treatment (day hospital vs. TAU) and primary outcomes at 18-month data were analyzed using MANCOVA, with age, gender and baseline metabolic features and baseline BMI as covariates. The associations of type of treatment and psychopathology at 18-month data were analyzed using MANCOVA, with age, gender and baseline PANSS-positive, PANSS-negative, PANSS-general as covariates. The type of treatment: day hospital vs. TAU, was not significantly associated with any of the primary or secondary outcome measures, after the adjustments, as follows BMI [*F*_(1, 38)_ = 0.00; *p* = 0.959; η^2^ = 0.00], triglycerides [*F*_(1, 38)_ = 0.64; *p* = 0.429, η^2^ = 0.017], cholesterol [*F*_(1, 38)_ = 2.29; *p* = 0.138, η^2^ = 0.057], LDL cholesterol [*F*_(1, 38)_ = 2.72; *p* = 0.107; η^2^ = 0.067], HDL cholesterol [*F*_(1, 38)_ = 0.00; *p* = 0.975; η^2^ = 0.00], glucose in blood [*F*_(1, 38)_ = 0.46; *p* = 0.504; η^2^ = 0.012]; PANSS-positive [*F*_(1, 63)_ = 2.17; *p* = 0.146; η^2^ = 0.03]; PANSS-negative [*F*_(1, 63)_ = 0.03; *p* = 0.86; η^2^ = 0.00]; PANSS-general [*F*_(1, 63)_ = 0.57; *p* = 0.454; η^2^ = 0.00] (Table 2).

**Table 2:** Changes of the primary and secondary outcomes according to the treatment group during the 18 months follow up.

	**Baseline**		**At 18th month**		**Absolute changes (95% CI)**		**Relative changes (%) (95% CI)**	
	**Day hospital (*n* = 21)**	**TAU (*n* = 50)**	**Day hospital (*n* = 21)**	**TAU (*n* = 50)**	**Day hospital (*n* = 21)**	**TAU (*n* = 50)**	**Day hospital (*n* = 21)**	**TAU (*n* = 50)**
**PRIMARY OUTCOME**								
BMI (kg/m^2^)	23 (3.0)	24 (3.6)	24 (3.7)	25 (4.2)	0.91 (−0.02 to 2.16)	2.14 (1.13 to 3.31)	4 (0 to 9)	9 (5 to 14)
Triglycerides (mmol/l)	1.3 (0.69)	1.3 (0.62)	1.7 (2.24)	1.4 (0.82)	0.66 (−0.33 to 2.41)	0.17 (−0.14 to 0.43)	98 (−18 to 331)	21 (−3 to 43)
Total cholesterol (mmol/l)	4.3 (0.56)	4.7 (0.99)	4.9 (0.94)	4.7 (0.92)	0.49 (0.06 to 1.02)	0.05 (–.41 to 0.46)	11 (1 to 23)	4 (−5 to 14)
HDL cholesterol (mmol/l)	1.3 (0.33)	1.5 (0.39)	1.4 (0.41)	1.4 (0.45)	0.07 (−0.03 to 0.18)	−0.03 (−0.14 to 0.07)	6 (−2 to 15)	−1 (−8 to 6)
LDL cholesterol (mmol/l)	2.4 (0.49)	2.6 (0.91)	3.0 (0.94)	2.7 (0.84)	0.40 (−0.04 to 0.91)	0.03 (−0.33 to 0.39)	14 (−21 to 36)	9 (−5 to 23)
Blood glucose (mmol/l)	5.4 (2.77)	4.9 (0.61)	4.9 (0.81)	5.4 (1.13)	−0.92 (−3.53 to 0.61)	0.65 (0.21 to 1.16)	−2 (−21 to 14)	14 (5 to 24)
**SECONDARY OUTCOMES**								
PANSS-positive	25 (8.5)	25 (8.9)	9 (3.99)	11 (7.2)	−15.3 (−21 to −9.57)	−15.4 (−19.4 to −11.1)	−58 (−71 to−42)	−55 (−67 to −43)
PANSS-negative	24 (7.2)	24 (7.6)	13 (4.86)	14 (6.6)	−10.5 (−12.7 to −7.8)	−11.5 (−14.2 to−8.6)	−46 (−54 to −35)	−45 (−54 to −35)
PANSS-general	50(11.3)	51 (10.5)	24 (7.6)	26 (10.3)	−25.2 (−31 to −19.1)	−28.9 (−33 to −24.3)	−53 (−61 to −44)	−56 (−61 to −49)
GAF, median (IQR)	20 (15 to 35)	23 (15 to 30)	85 (80 to 90)	85 (63.8 to 91.2)	58.2 (46.7 to 68.9)	57.4 (52 to 62.6)	314 (189 to448)	283 (216 to 359)

Data are presented as mean (standard deviation) if not stated otherwise.

*CI, confidence interval; IQR, interquartile range*.

## Discussion

We have assessed the effects of a multimodal psychosocial intervention on the antipsychotic-induced weight gain and metabolic changes in a moderate sample of first episode psychosis over a longer period. Overall, we did not confirm the hypothesis that a general multimodal program is more effective in counteracting weight gain or antipsychotic-induced metabolic abnormalities compared to TAU in patients with first episode psychosis over a longer follow-up period. In fact, both approaches seem to be rather effective for restoring patients' level of functioning, but less effective to counteract antipsychotic-induced weight gain and metabolic abnormalities, as the increase in body mass index and metabolic changes are significant over 18-months.

Unfortunately, this is in line with other studies which found that different interventions (lifestyle, psychoeducation, exercise) are of limited effectiveness when it comes to antipsychotic-induced weight gain or metabolic changes over a longer time period (e.g., 18-months of antipsychotic-treatment) (19, 20). This is also in line with the results of our previous study performed at the ZUHC, where we found no effects of the PsyLOG m-health intervention compared to TAUin preventing antipsychotic-induced weight gain and metabolic changes over 6 months follow-up (22). However, it is also possible that patients with first episode of psychosis are far more vulnerable group compared to patients with multiple episodes of psychosis when it comes to antipsychotic-induced weight gain. In fact, these patients usually have several risk factors for significant antipsychotic-induced weight gain such as lower initial body weight, better treatment response and first treatment with antipsychotics. Furthermore, while being correlated to a certain extent, some of these antipsychotic-induced metabolic abnormalities, such as diabetes, often occur independently of weight gain, and thus can be counteracted only by close monitoring (23).

We did not analyze the effect of medication thoroughly. As most of the patients were treated with polypharmacy at different stages of treatment it is difficult to assess the effect of medication on weight gain and metabolic changes in this study. Although the majority of guidelines suggest monotherapy in the treatment of psychosis, polypharmacy is widespread in psychiatric practice worldwide (4), including the region where this study was performed (24). Thus, it is probably not sufficient to recommend monotherapy as the main strategy to counteract antipsychotic-induced weight gain and metabolic abnormalities as it is fair to assume that clinicians probably decide to use polypharmacy to treat complex clinical symptoms. The relatively high levels of functioning and significant decrease of the severity of psychopathology in these patients after 18-months of treatment suggest that effectiveness of treatment in terms of functioning is the main goal of treatment, while antipsychotic-induced weight gain or metabolic changes are regarded as a less important at best. However, acknowledging the effect of metabolic abnormalities on morbidity, more focused strategies to counteract these side effects are needed. While several interventions involving exercise proved to be efficient over a shorter period, this effect is lost over a longer period (19, 20). As the negative effects of metabolic abnormalities became apparent over time, it is crucial to sustain the effects of these interventions, possibly with a combination with psychosocial or behavioral interventions focused on sustaining motivation to exercise or regular supervision of exercise over a longer period (25). Therefore, finding a suitable program that could help sustaining the motivation for the continuation of exercises over a longer period still remains a treatment challenge.

## Limitations of the study

First, we did not use a randomized controlled design to assign the patients to the assessment groups, but we used a naturalistic design. While randomized control design is considered as standard for assessing the efficacy of an intervention, naturalistic design may mirror the real-life situation better. Furthermore, it could be argued that since the patients were choosing themselves one the two treatment options (day hospital or TAU) there may be systematic difference between them in aspects not measured in the study, e.g., motivation, adherence, etc. which may influence metabolic outcomes. Finally, we cannot exclude the effects of other factors not included in the study, such as personality styles, eating habits and levels of activity during the studied period on metabolic outcomes.

## Author contributions

MRK designed and performed the study, analyzed the data, and wrote the first draft of the study. DBK designed and performed the study, critically analyzed the data, and wrote the first draft of the study. IK and PM designed the study, analyzed the data and gave critical comments, and revised the first draft of the study. ZM, AKM, SKM, PB, DBK, and ZV performed the study and gave critical comments and revised the first draft of the study. ZB analyzed the data and gave critical comments and revised the first draft of the study.

### Conflict of interest statement

The authors declare that the research was conducted in the absence of any commercial or financial relationships that could be construed as a potential conflict of interest.
